# Potential Use of Brazilian Green Propolis Extracts as New Photosensitizers for Antimicrobial Photodynamic Therapy against Cariogenic Microorganisms

**DOI:** 10.3390/pathogens12020155

**Published:** 2023-01-17

**Authors:** Analú Barros de Oliveira, Túlio Morandin Ferrisse, Gabriela Gomes França, Sarah Raquel de Annunzio, Willian Kopp, Carla Raquel Fontana, Fernanda Lourenção Brighenti

**Affiliations:** 1 Department of Morphology and Pediatric Dentistry, School of Dentistry, São Paulo State University (UNESP), Araraquara 14801-903, SP, Brazil; 2 Department of Dental Materials and Prosthodontics, School of Dentistry, São Paulo State University (UNESP), Araraquara 14801-903, SP, Brazil; 3 Department of Clinical Analysis, School of Pharmaceutical Sciences, São Paulo State University (UNESP), Araraquara 14800-903, SP, Brazil

**Keywords:** antimicrobial photodynamic therapy, natural photosensitizer, Brazilian green propolis, cariogenic microorganisms

## Abstract

The synergic effect of *Streptococcus mutans* and *Candida albicans* increases dental caries severity. Antimicrobial photodynamic therapy (aPDT) is a non-invasive treatment for antimicrobial aspects. However, the current photosensitizers (PS) have many downsides for dental applications. This study aimed to evaluate the efficiency of two different Brazilian green propolis (BGP-AF and BGP-AG) as PS for aPDT against these microorganisms. A single-species biofilm was irradiated with crude extracts and their fractions and controls. Such extracts showed the best results and were evaluated in dual-species biofilms. Photodegradation, reactive oxygen species (ROS), cytotoxicity, and color stability assays were also investigated. Reductions higher than 3 log_10_ CFU/mL (*p* < 0.0001) occurred for crude BGP in single- and dual-species biofilms. Singlet oxygen was produced in BGP (*p* < 0.0001). BGP-mediated aPDT delayed *S. mutans* and *C. albicans* regrowth after 24 h of treatment (*p* < 0.0001). Both BGP did not change the color of dental materials (*p* > 0.05). BGP-AF-mediated aPDT showed 72.41% of oral keratinocyte viability (*p* < 0.0001). BGP extracts may be used in aPDT against *S. mutans* and *C. albicans*. Specifically, BGP-AF may represent a promising PS for dental applications.

## 1. Introduction

Dental caries is among the most prevalent chronic diseases in the world, affecting 48% of children, causing life-long consequences, and harming the quality of life of people [1]. Dental biofilm is an important etiological factor of dental caries. Consequently, mechanical removal by brushing associated with fluoride toothpaste and lower sugar consumption are the traditional methods for controlling and preventing this disease [2]. However, tooth brushing effectiveness depends on the individual’s motor ability and sometimes requires adjunctive therapies, such as the chemical control of dental biofilm [3]. Chlorhexidine usually controls dental biofilms, but its prolonged use may cause undesirable side effects [4].

Although *Streptococcus mutans* is a relevant cariogenic bacterium, dental caries is a multifactorial disease involving complex interactions between genetic, microbial, biochemical, biophysical, and environmental factors [5,6]. Additionally, the association of *S. mutans* with *Candida albicans* may increase *S. mutans* pathogenicity by upregulating the virulence factor of dental biofilms, which will affect dental caries establishment and severity [7,8].

Several studies have demonstrated the efficacy of photodynamic therapy (PDT) in cariogenic bacteria [9,10,11], highlighting that it may help control dental caries [12,13]. PDT is a non-invasive therapy that requires specific light sources, the presence of oxygen, and a photosensitizing agent (PS) to cause cell death [14]. Thus, a PS must be available in its pure form, displaying its chemical composition. Moreover, an acceptable PS should present the following characteristics: (i) synthesizability from available and easily reproduced precursors, favoring singlet oxygen production; (ii) photo- and thermodynamic stability; (iii) selective induction of target cell death and biological activity only when exposed to the light source; (iv) stability and solubility in body tissue fluids; (v) convenient delivery to target tissues through injection or other methods; (vi) easy elimination after finishing the treatment [15].

However, most available PS have numerous limitations, such as low cell permeability, a different absorption band from the desired therapeutic window, long permanence in some tissues, toxic secondary metabolite formation after irradiation, and limited antimicrobial capacity. Thus, the number of scientific publications using natural products as PS has recently increased [16,17,18,19]. This may be due to the ability of these substances to adhere to or cross the cytoplasmic membrane and their potential for reactive oxygen species production [20]. Therefore, studies on natural materials can be a compelling source for discovering photosensitizing agents for PDT.

Brazilian green propolis (BGP) extract presents several biological activities, such as anti-tumor, antibacterial, anti-inflammatory, anti-hypertensive, antioxidant effects, and biocompatibility [21,22,23,24]. However, only one study evaluated BGP as a PS in PDT for treating squamous cell carcinoma, showing promising results [25].

Despite favorable data on the antibacterial properties of BGP, the literature does not report the photosensitizing potential of this extract in aPDT. Thus, this study aimed to evaluate the in vitro potential of two commercial BGP extracts and their fractions as PS for aPDT application in single- and dual-species biofilms of *S. mutans* and *C. albicans*. It also evaluated photodegradation, reactive oxygen species (ROS) quantification, cytotoxicity, and color stability of BGP extracts.

## 2. Materials and Methods

### 2.1. Obtaining Brazilian Green Propolis Extracts

The Brazilian green propolis (BGP) included in this study was registered with the Ministry of Environment (“SISGEN”; registration number: AFC82A9). The samples were obtained from Apis Global Produtos Alternativos Ltd.a™ (BGP-AG) and Apis Flora Industrial e Comercial Ltd.a™ (BGP-AF). Both BGP extracts are ethanol based. The raw materials were collected in São Paulo, Brazil, according to the manufacturers. The same batch of each extract was used throughout the experiment. The substances were stored according to the manufacturers’ instructions. The BGP extracts were diluted in a phosphate buffer solution (PBS—0.1 M; pH: 7.2) at a 1:50 ratio (BGP: culture medium) and tested at a 1% concentration. All BGP solutions were freshly prepared in a light-protected environment. The two standardized crude extracts offer advantages over other marketed products because some of their biological activities and biocompatibility have been studied [26,27], allowing higher safety for the present study.

### 2.2. Extraction of Water-Soluble Substances

Water-soluble substances were extracted using 4.0 g of BGP-AF and BGP–AG. The extracts were weighed and dissolved in 36 mL of ethanol, producing a 10% solution (*w/v*). The resulting solutions remained under mechanical stirring for 16 h. Next, the subsequent blends were centrifuged at 4500× *g* for 10 min in an ultracentrifuge (Eppendorf, Germany) for sedimenting the insoluble fraction. Finally, the soluble fraction was carefully separated with a Pasteur pipette and stored, at 4 °C, until reconstitution.

Ethanol was evaporated under decreased pressure (60 °C; −250 mmHg) up to a volume lower than 5 mL. Then, 50 mL of Type II water was added, and the procedure was repeated three times. At the end of the method, the extracts were reconstituted in water by adding 50 mL of Type II water and stored, at 4 °C.

The extracts were analyzed for their interaction with light in the 200–800 nm range using a UV-Vis spectrophotometer with a DAD detector (Analitk Jenna, Germany). Hence, the extracts were diluted in Type II deionized water or ethanol and scanned with a quartz cuvette. For the reconstituted samples, Type II water was used to record the baseline and ethanol for the ethanol samples.

Protein concentration in the reconstituted extracts was assessed with the Bradford method [28]. A commercial Bradford solution (Sigma Aldrich, Saint Louis, MO, USA) was used in the experiments. Microtubes of 1.5 mL received 1 mL of commercial Bradford solution and 25 μL of extract solution. After a five-minute incubation, at room temperature, a spectrophotometer (Analitk Jenna, Germany) determined absorbance (λ = 595 nm). The total protein concentration was then calculated from the signal generated at 595 nm using a calibration curve previously constructed with Bovine Serum Albumin (BSA).

#### Fractionation of Crude Extracts

The crude extracts were fractionated to assess whether the photodynamic activity of fractions improves or remains constant relative to the crude extracts. Considering that Brazilian green propolis is a mixture of several substances, it is not easy to extract or obtain fractions. The extraction method was the alcohol-soluble fraction, called resin fraction. This process also produces the alcohol-insoluble fraction, forming the waxy fraction.

### 2.3. Light Sources

The visible light absorption spectrum was measured with a microplate fluorescence reader (Synergy H1a Multi Mode Reader, Biotek, Winooski, VT, USA). PDT was performed with IrradLED™—Biopdi (São Carlos, SP, Brazil), equipped with 48 LEDs and a cooling system to prevent overheating. Pilot studies were conducted to set the best parameters (irradiance dose, intensity, and concentration of plant material; data not shown). The best bacterial reduction occurred at 450 nm (151 mW/cm^2^) and fractionated mode every 60 s.

### 2.4. Photodegradation Assay

The test used extracts at 0.25% and an irradiance dose of 80 J/cm^2^ (154 mW/cm^2^). Total irradiation time was divided into five groups: 103, 206, 309, 412, and 519 s. The readings were performed in 100 μL of the solutions added to 96-well microplates using a SynergyH1 Multi-Mode Reader (BioTek, Winooski, VT, USA).

### 2.5. Bacterial Strain and Growth Conditions

The reference strains of *Streptococcus mutans* ATCC™ UA159 and *Candida albicans* ATCC™ 90028 were obtained from the Oswaldo Cruz Foundation (FIOCRUZ) collection and maintained at −80 °C.

The strains were thawed and seeded individually using the quadrant depletion technique in one of the following solid culture media: Sabouraud Dextrose Agar with 5 mg/mL of chloramphenicol (SDA, Acumedia Manufacturers Inc., Baltimore, MD, USA) for *C. albicans* (incubation, at 37 °C, in an aerobic oven for 48 h) and Brain Heart Infusion (BHI) (Difco, Laboratories, Detroit, MI, USA) supplemented with 1% sucrose and 0.2 IU/mL of amphotericin B (AmB) for *S. mutans* (incubation in 5% CO_2_, at 37 °C, for the same time).

For pre-inoculum preparation, five colonies of *C. albicans* and ten colonies of *S. mutans* were inoculated with a sterile disposable loop in 10 mL of Yeast Nitrogen Base (YNB) or BHI broth with 1% glucose, respectively. The microorganisms were incubated overnight (16–18 h), at 37 °C, in aerobiosis (*C. albicans*) or 5% CO_2_ (*S. mutans*).

Next, the pre-inoculum was diluted at two ratios (1:10 and 1:20) to prepare the inoculum. For the 1:20 dilution ratio, 500 μL of the pre-inoculum was added to 9.5 mL of culture media for each microorganism. In the 1:10 dilution ratio, 1000 μL of the pre-inoculum was added to 9.0 mL of culture media. The optical density of dilutions (λ = 540 nm for *C. albicans* and λ = 600 nm for *S. mutans*) was assessed. The tubes were incubated in aerobiosis, at 37 °C (*C. albicans*), and in a 5% CO_2_ oven, at 37 °C (*S. mutans*), until their mid-log phases (A_540_ = 0.7 ± 0.01 for yeast and A_600_ = 0.7 ± 0.01 for bacteria), representing a microbial concentration of 10^7^ CFU/mL of each microorganism. According to pre-established growth curves, this optical density was reached after incubation for approximately four and eight hours for *S. mutans* and *C. albicans*, respectively.

### 2.6. Antimicrobial Photodynamic Therapy (aPDT) on Single-Species Biofilm

The experiments occurred on two separate occasions (n = 10) and in duplicates with the same sample size. Hence, the mean was calculated from the duplicate experiments to allow group comparisons.

Single-species biofilms were grown in 96-well plates, according to Fontana et al. [29]. Microbial suspensions of *S. mutans* or *C. albicans* were prepared separately, as previously described (Section 2.5). The wells received 150 µL of the inoculum, and plates were incubated according to the parameters described in Section 2.5 for three days. In the first 24 h, the culture media were not refreshed. After the second day, the spent media were removed and received 150 µL/well of fresh broth. The culture media were also refreshed on the third day of biofilm growth when the biofilm matured, and aPDT was performed. This step tested BGP crude extracts and their fractions. Therefore, each well containing the mature biofilm received 50 µL of 1% BGP extract/fraction. After 15 min of incubation, each propolis extract received aPDT. Then, the biofilm was gently detached with 1 µL of inoculation loop and diluted for further cultivation and counting of colony-forming units. Table 1 describes the used parameters and experimental groups.

Ten groups were studied: (1) negative control (growth control), (2) BGP-AG crude, (3) BGP-AG fraction, (4) BGP-AF crude, (5) BGP-AF fraction, (6) PDT1 (light + BGP-AG crude), (7) PDT2 (light + BGP-AG fraction), (8) PDT3 (light + BGP-AF crude) (9) PDT4 (light + BGP-AF fraction), and (10) light exposure. Groups BGP and PDT were pre-incubated with BGP extracts (crude or fraction) for 15 min in the dark and at room temperature. Light and negative control groups were pre-incubated in a culture medium for 15 min in the dark and at room temperature. Irradiation (bottom-up light) occurred in Light and PDT groups at 450 nm and 80 J/cm^2^ (Table 1). The irradiation dose was calculated from the light dose formula (J/cm^2^) = I (W/cm^2^) × t (sec), resulting in 18 min of fractional irradiation mode. Each plate received only one treatment to prevent any interference during irradiation. BGP and negative control groups were not irradiated and remained at room temperature for 18 min.

After the treatment, the suspensions were diluted, and 5 µL of each dilution was plated with the agar drop method [30]. After 48 h of incubation under the conditions described in item 2.5, a blind examiner determined the number of colony-forming units per milliliter (CFU/mL).

### 2.7. Antimicrobial Photodynamic Therapy (aPDT) on Dual-Species Biofilm

This step evaluated, using the same experimental parameters, only the groups that showed the best results in reducing the microbial load for *S. mutans* and *C. albicans* dual-species biofilms (Table 1). Microbial viability was evaluated on two occasions: (i) immediately after treatment and (ii) after regrowth for 24 h [31].

After adjusting the concentrations, 50 µL of *C. albicans* and *S. mutans* suspensions were transferred to 96-well plates (100 µL/well). The plates were then incubated in a 5% CO_2_ incubator, at 37 °C, for 90 min, corresponding to the biofilm adhesion phase. Next, the wells were washed twice with sterile PBS (0.1 M; pH: 7.2). Then, BHI broth supplemented with 1% glucose was added, and the biofilms were incubated in 5% CO_2_, at 37 °C. After 24 h, 50 µL of the spent culture medium was removed, and the biofilms received the same volume of fresh culture medium. The plates returned to the incubator for another 24 h to complete 48 h of biofilm formation. After this period, the biofilm received the treatments as described in Section 2.6.

Six groups were evaluated at this stage for each microorganism: negative control (growth control), BGP-AG (crude), BGP-AF (crude), PDT1 (light + BGP-AG extract (crude)), PDT2 (light + BGP-AF extract (crude)), and light exposure.

After the treatments, the biofilms were washed twice with PBS (1M; pH 7.2) and carefully scraped for 30 s to detach them from the bottom of the well. Serial dilutions (10^−1^, 10^−2^, 10^−3^, 10^−4^, and 10^−5^) were prepared as follows: 100 μL of the dispersed biofilms solution was transferred to a microtube containing 900 μL of PBS to prepare the 10^−1^ dilution. The 10^−2^ dilution was prepared by transferring 100 μL of the 10^−1^ dilution to 900 μL of PBS. This was repeated until obtaining the 10^−5^ dilution. Then, 5 µL from each concentration was seeded in BHI agar supplemented with amphotericin and SDA agar supplemented with chloramphenicol, for CFU assessment of *S. mutans* and *C. albicans*, respectively. The agar plates were incubated under aerobic conditions, at 37 °C (±1 °C), for 48 h. Next, the colonies were counted, and microbial concentration was expressed as CFU/mL and calculated as a logarithm scale (log_10_).

Biofilms from the regrowth group were reincubated in 100 µL of BHI supplemented with 1% sucrose and 100 µL Sabouraud liquid medium for additional 24 h, as described in Section 2.5, and then detached and dispersed as mentioned above.

### 2.8. Reactive Oxygen Species (ROS) Detection Cell-Free System (Solution)

The 3′-p-(aminophenyl) fluorescein and Singlet Oxygen Sensor Green (Invitrogen, Thermo Fischer Scientific, Waltham, MA, USA) fluorescent probes measured hydroxyl radicals [•OH] and singlet oxygen, respectively. Solutions containing 1% BGP extracts were prepared in PBS (0.1 M; pH 7.2) and mixed with 3 µmol/mL (final concentration) of the probes in dark flat-bottom 96-well plates (Corning™). Irradiation occurred as previously described. The Synergy H1M (BioTek, Winooski, VT, USA) assessed the readings. Excitation/emission wavelengths were 490/515 nm for 3′-p-(aminophenyl) fluorescein and 505/525 nm for Singlet Oxygen Sensor Green.

### 2.9. Cytotoxicity Assessment

Cells from oral keratinocyte lines (NOK-SI) were cultured with 75 cm^2^ cell culture flasks and incubated, at 37 °C, in 5% CO_2_ (MCO-17AC, Sanyo Electric Co., Ltd., Osaka, Japan). The DMEM (Dulbecco’s Modified Eagle’s Medium, Dulbecco’s Modified Eagle’s Medium, Campinas, SP, Brazil) supplemented with 10% fetal bovine serum (Cultilab, Campinas, SP, Brazil) was renewed every 48 h.

Extracts at 1% BGP were prepared in DMEM. Triton X-100 corresponded to the dead control. DMEM supplemented with 10% SFB represented the live control. The cells were trypsinized after reaching 80% confluence. The suspension containing 1.7 × 10^5^ cells/mL of each cell line was seeded in 96-well plates and incubated for 24 h in 5% CO_2_ [32]. Then, the culture media were renewed, and the cells were treated with BGP extracts and controls for 15 min of pre-incubation and then 18 min of irradiation, as described in Section 2.6.

Next, the culture medium was removed and received 100 μL of MTT (3 mg/mL, Sigma Aldrich). After three hours, the suspension was removed, and absorbance (λ = 562 nm) was assessed. The experiments were performed in triplicate, and the cell viability percentage was calculated with the negative control as 100% viability [33]. 

### 2.10. Effect of BGP-Mediated Antimicrobial Photodynamic Therapy on the Color Stability of Restorative Dental Materials

#### 2.10.1. Preparation of Specimens

The specimens (n = 12) were prepared using glass ionomer cement (GIC) Ketac Molar EasyMix (color A3) (3M ESPE, Campinas, SP, Brazil) and composite resin Z250 (color A3) (3M ESPE, Campinas, SP, Brazil). GIC was prepared at room temperature according to the manufacturer’s instructions.

GIC was inserted into matrices (3 mm height × 6 mm ø) and a 1 mm-thick polyester strip weighing 100 g, for 30 s, for planning and eliminating excess material [34]. Composite resin specimens were prepared with the incremental technique. The specimens were light-activated (20 s; VALO Cordless; Ultradent, South Jordan, UT, USA; radiant emittance 1100 mW/cm^2^, wavelength between 395 and 400 nm) [35].

#### 2.10.2. Color Stability

Color stability of the samples (n = 12) was determined with the BYK-Gardner color guide spectrometer, according to the *Commission Internationale de l’Eclairage* (CIE) L*a*b* system [36]. All assessments were performed three times, calculating the mean values of L*, a*, and b*. The total color change (ΔE) of each specimen was calculated with **ΔE** = ½ [(**L**1 − **L**0)^2^ + (**a**1 − **a**0)^2^ + (**b**1 − **b**0)^2^]. The ΔE is the difference between two times—before and after light application. The specimens of groups not exposed to light (PDT) remained in contact with the PS or PBS (control) for the same time of light exposure in the PDT group.

### 2.11. Statistical Analysis

The sample size was calculated with Bioestat 5.0 after a pilot study and with the following parameters: the minimum difference between means (0.0186), standard deviation (0.0243), the number of repetitions, power of analysis = 0.80, and α = 0.05 (n = 10). The data were analyzed with IBM SPSS 20.0. A descriptive data analysis assessed the results, followed by normal distribution and homoscedasticity verification with the Shapiro–Wilk and Levene tests, respectively. After verifying these parameters, a two-way analysis of variance (two-way ANOVA) was conducted. For microbial viability in the single species, the light parameters and type of BGP (crude and extracts) represented the independent variables. Light parameters and microbial evaluation immediately and 24 h post-treatment corresponded to the independent variables for microbial viability in the dual-species. As for ROS detection, the BGP extracts and fluorescent probes were the independent factors. The presence and absence of light and the different evaluated groups represented the independent parameters for cytotoxicity.

After two-way ANOVA, the means were estimated at a 95% confidence interval for multiple comparisons. Additionally, Pearson’s correlation was performed between the probe in ROS detection and the BGP extracts. Hence, Pearson’s correlation coefficient was calculated. Finally, photodegradation and color stability were assessed with the Kruskal–Wallis test following Dunn’s test to identify significant differences among the groups. The statistician was blinded in all tests.

## 3. Results

### 3.1. Physicochemical Characterization of Brazilian Green propolis Extracts and Preparation of the Crude Extract Fraction

**Figure 1:**
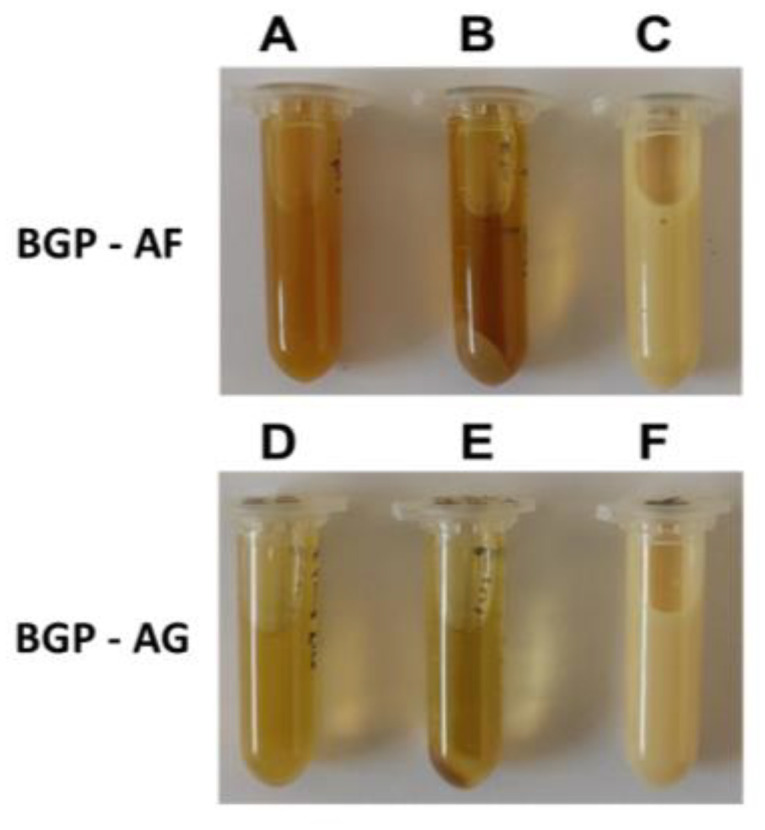
Samples prepared during the study. BGP-AF: (**A**) Ethanol extract; (**B**) Centrifuged ethanol extract; (**C**) Extract reconstituted in water. BGP-AG: (**D**) Ethanol extract; (**E**) Centrifuged ethanol extract; (**F**) Extract reconstituted in water.

Extraction in ethanol was performed in the first step, producing solutions with slightly different colors, and the BGP-AF extract was the darkest (Figure 1). Centrifugation allowed the separation of a significant amount of sediment for both extracts. The BGP-AF extract produced a higher sediment concentration than BGP-AG.

After extraction with ethanol, propolis component substances were reconstituted in water to eliminate the ethanol and obtain an aqueous solution [37]. This solution provides a chemical environment similar to that of photosensitization procedures, allowing the accurate characterization of the light interaction profile of these substances. The reconstitution procedure considerably changed the appearance of both extracts, as seen in Figure 1. The originally translucent aspect becomes yellowish and milky, probably due to the low solubility of some substances in water. The mixture is likely to form an emulsion, indicating that some components have surfactant features, possibly from fatty acids and/or oligosaccharides [38].

The two reconstitution cycles eliminated most residual ethanol in the extract. After the procedure, the propolis concentration in both extracts was lower than 10% (m/m) due to the loss of insoluble substances during sedimentation by ultracentrifugation. These procedures generated only one fraction for each Brazilian green propolis extract.

#### Spectrophotometric Profile of the Extracts

**Figure 2:**
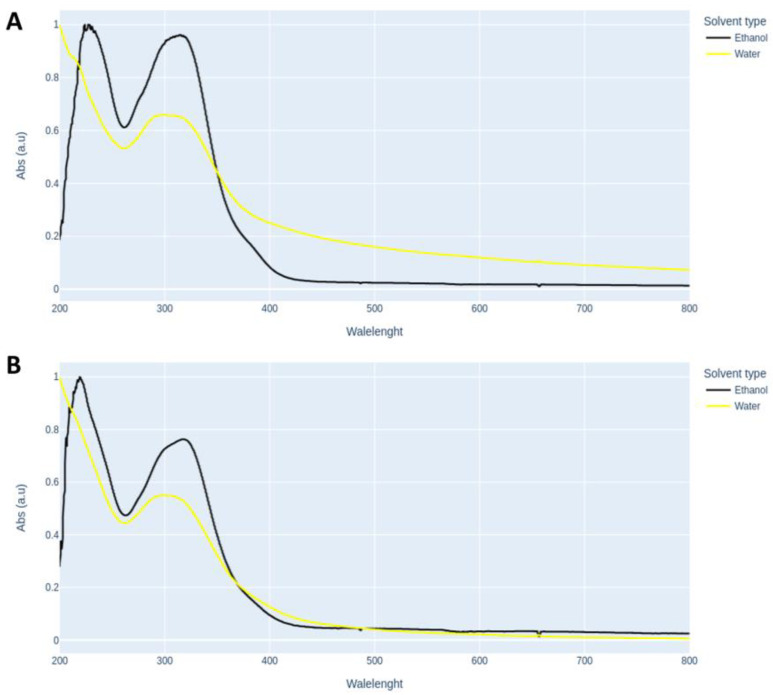
Spectrophotometric profile of BGP extracts (**A**) BGP-AF (Apis Flora) and (**B**) BGP-AG (Apis Global). The spectrophotometric profile was recorded in the aqueous or ethanolic solutions of the respective extracts diluted 400× (final concentration = 0.004%). The scan was performed using an Analytik Jena spectrophotometer (Germany) equipped with a DAD detector.

The extracts obtained in this study (ethanolic and aqueous) were analyzed for their interaction with light in the UV-Vis (200–800 nm). The concentration of substances capable of interacting with light in the extracts was extremely high, requiring a 400× dilution (final concentration = 0.004%) to effectively evaluate the spectrophotometric profile of the extracts (Figure 2).

Moreover, this study directly compared the signal at 450 nm for the samples (Table 2) and the total protein concentration in ethanolic propolis extracts reconstituted in water and measured with the colorimetric method by Bradford (Table 3).

### 3.2. Visible Light Absorption Spectrum

**Figure 3:**
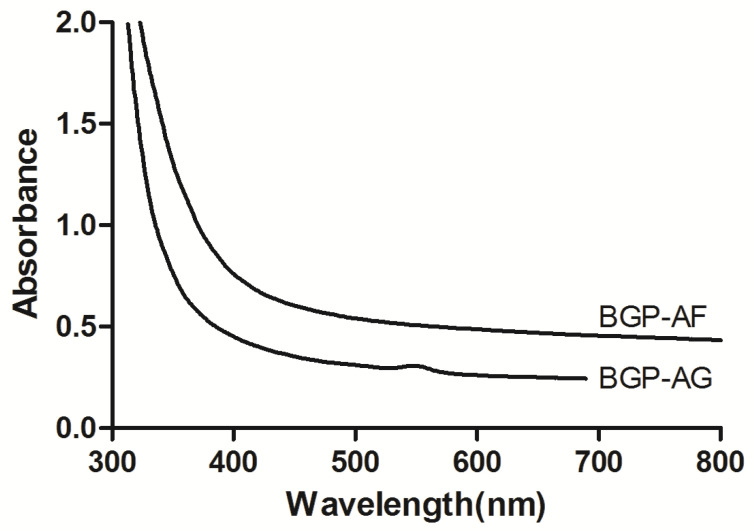
Absorption spectrum graphs of Brazilian green propolis at 1% concentration diluted in phosphate-buffered saline (PBS—0.1 M; pH: 7.2).

The analysis of both BGP extracts revealed the main absorption band corresponding to the most frequent light source in dental offices and hospitals. The spectrum of interest (400–450 nm) showed light absorption, indicating a potential application of these BGP extracts in aPDT. Figure 3 shows the absorption spectra of BGP extracts.

### 3.3. Photodegradation

**Figure 4:**
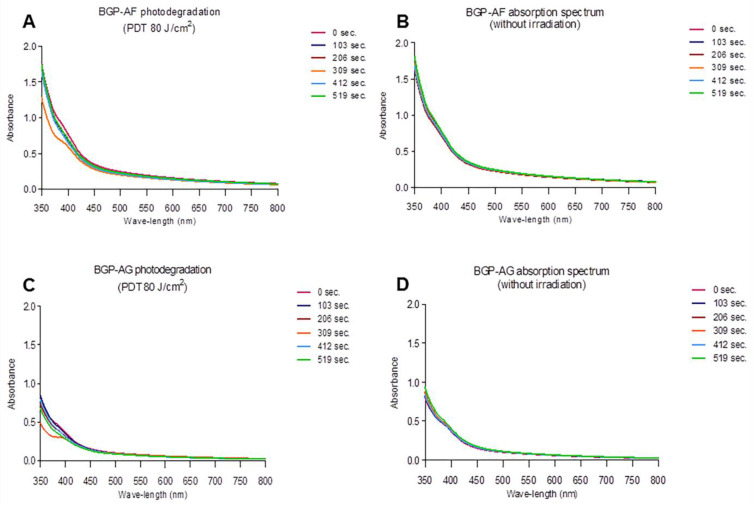
Photodegradation kinetics of BGP-AF at a 0.25% concentration (**A**); Photodegradation kinetics of BGP-AG at a 0.25% concentration (**B**); Light absorption spectrum of BGP-AF in the absence of light (**C**); Light absorption spectrum of BGP-AG in the absence of light (**D**).

Both extracts showed a slight decrease in absorbance values after the irradiation times, but this difference was not significant (*p* > 0.05). Despite the reduction in absorbance values, the extracts showed absorption in the wavelength spectrum between 400 and 450 nm, even after 519 s of irradiation, showing that the molecules remained available during irradiation. Additionally, there was no formation of new absorption bands at other wavelengths (Figure 4A–D).

### 3.4. Antimicrobial Photodynamic Therapy (aPDT) on Single-Species Biofilm

**Figure 5:**
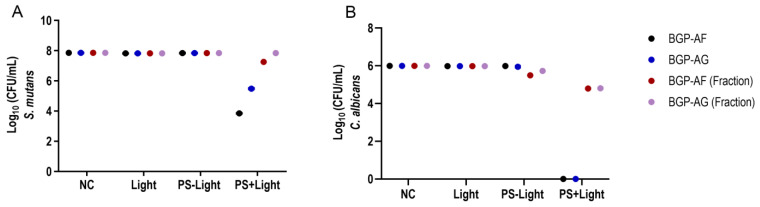
aPDT effect of 1% BGP extracts (crude/fraction) on microorganisms in the single-species biofilm of *S. mutans* (**A**) and *C. albicans* (**B**). Two-way ANOVA (mean ± 95% confidence interval; n = 10). NC = negative control; PS-light = BGP without exposure to light; PS + light = BGP with exposure to light; Light: only exposure to light.

BGP-AF (5.05 log_10_ CFU/mL) and BGP-AG (3.04 log_10_ CFU/mL) extracts showed a significant reduction in *S. mutans* biofilm viability when exposed to light (PS + Light) (Figure 5A). BGP fractions presented only a slight microbial reduction when exposed to light. *C. albicans* biofilm viability had similar results: a total reduction (6.0 log_10_ CFU/mL) of microbial load for BGP crude extracts exposed to light (PS + Light) and a slight reduction in microbial viability for BGP fractions (Figure 5B). BGP crude extracts and fractions could not reduce the microbial load in the absence of light (PS-Light) (Figure 5A,B). Regarding both microorganisms evaluated, only light exposure (Light) could not reduce microbial load. Table 4 shows the statistical details.

### 3.5. Antimicrobial Photodynamic Therapy (aPDT) on Dual-Species Biofilm

**Figure 6:**
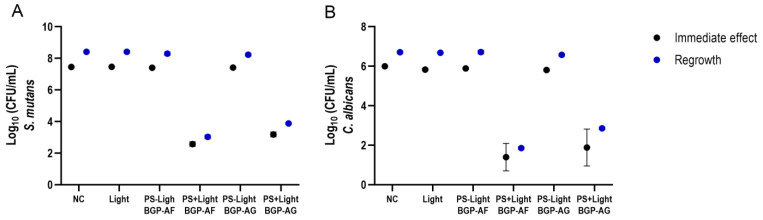
aPDT effect of 1% BGP crude extracts on dual-species biofilm viability (*S. mutans* (**A**) and *C. albicans* (**B**)). Two-way ANOVA (mean ± 95% confidence interval; n = 10). NC: negative control; PS-light = BGP without exposure to light; PS + light = BGP with exposure to light; Light: only exposure to light.

Considering that the BGP fraction results did not substantially reduce microbial viability in single-species biofilms, only BGP crude extracts participated in the dual-species experiment. For *S. mutans,* BGP extracts highly reduced microbial viability immediately after exposure to light (PS + Light) (BGP-AF = 4.89 log_10_ CFU/mL/ BGP-AG = 4.27 log_10_). Advantageously, 24 h after treatment (PS + Light), both extracts still showed a suitable decrease in *S. mutans* regrowth ability (BGP-AF = 5.38 log_10_ CFU/ mL/ BGP-AG = 4.53 log_10_ CFU/mL) (Figure 6A). There were similar results for *C. albicans* viability when exposed to light (PS + Light) (immediate effect: BGP-AF = 4.60 log_10_ CFU/mL/ BGP-AG = 4.11 log_10_; regrowth: BGP-AF = 4.86 log_10_ CFU/mL/BGP-AG = 3.86 log_10_ CFU/mL) (Figure 6B). BGP-AF (PS + Light) showed the best results for both microorganisms. Table 4 shows further details about the statistical procedures.

### 3.6. Reactive Oxygen Species (ROS) Detection

**Figure 7:**
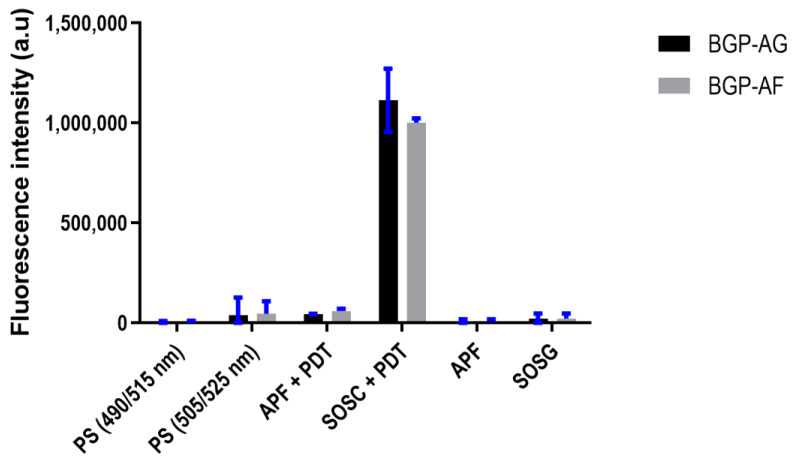
ROS production in BGP extracts after exposure to light (mean ± 95% confidence interval).

Both extracts showed a higher singlet oxygen production. Control experiments without PS resulted in negligible fluorescence values for both probes, suggesting that under these conditions, there was no reactive oxygen species production (Figure 7). Table 4 shows details about the statistical test.

### 3.7. Pearson’s Correlation

A Person’s correlation study was performed to understand the association between *S. mutans* and *C. albicans* reduction in dual-species biofilm and singlet oxygen production.

The correlation was significant (*p* < 0.05) for both BGP extracts, except for *C. albicans* regrowth. Moreover, the significant correlations could be classified as strong (<0.6; r;<0.9) and very strong (<0.9; r; <1) (Table 5).

### 3.8. Cytotoxicity Assessment

**Figure 8:**
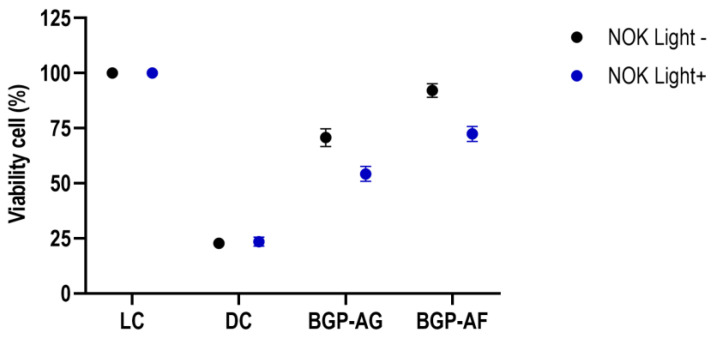
Cytotoxicity of 1% BGP crude extracts in the presence or absence of light. Two-way ANOVA (mean estimation at a 95% confidence interval; n = 16. LC= live control; DC = dead control; NOK Light− = NOK cells in the absence of light; NOK Light+ = NOK cells in the presence of light.

Both BGP crude extracts showed a significant difference between the live control (LC) and dead control (DC) in the absence of light. Although BGP crude extracts showed a significant reduction in oral keratinocyte viability after light application, both BGP extracts remained significantly different from LC and DC. BGP-AF without light presented higher viability (92.11%). After light application, group BGP-AF (72.41%) had better oral keratinocyte viability than BGP-AG (54.25%) (Figure 8). Table 4 provides further details about the statistical procedures.

### 3.9. Effect of BGP-Mediated Antimicrobial Photodynamic Therapy on the Color Stability of Restorative Dental Materials

**Figure 9:**
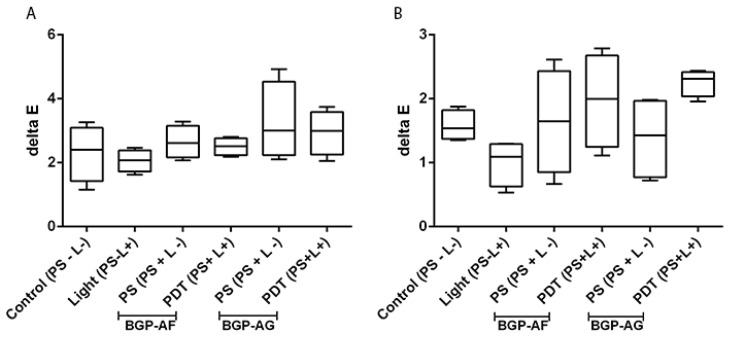
Effect of BGP-mediated aPDT on the color stability of composite resin (**A**) and glass ionomer (**B**). Kruskal–Wallis test. There were no significant results (*p* > 0.05) for both dental restorative materials. In the control group (PS − L−), only PBS was inserted into the plates with restorative dental materials. In the Light group, the restorative dental materials remained with PBS and received light. In the PS group, restorative dental materials remained with the PS and did not receive light. For the PDT group, restorative dental materials received PS and light.

GIC and composite resin did not show significant differences in the color stability assay (*p* > 0.05; Figure 9).

## 4. Discussion

To the best of our knowledge, this is the first time that Brazilian green propolis (BGP) has been evaluated as a photosensitizer (PS) in antimicrobial photodynamic therapy (aPDT). The present study showed that photoactivation of 1% BGP significantly reduced *S. mutans* and *C. albicans* viability in either single- or dual-species biofilm. Therefore, the antimicrobial activity of BGP extracts was effective against *S. mutans* and *C. albicans* biofilms even at low concentrations.

It is worth noting that the researcher who assessed microbial viability and the statistician were blinded. This is of utmost relevance because non-blinded studies can cause biases that may affect the outcome [39]. Thus, all blinding possibilities are appreciated due to the singularities of in vitro studies.

Both BGP crude extracts showed a microbial reduction in *S. mutans* and *C. albicans* higher than 3 log_10_ CFU/mL in single- and dual-species biofilms. Such a reduction might indicate that an antimicrobial compound will show clinical significance [40]. It is also noted that the used BGP extract concentration (1%) is below the maximum dose recommended for natural materials (3%) [41].

The interest in phytomedicine and natural compound properties for treating and controlling several diseases has increased over the past years. In this context, natural compounds may represent an alternative to classic PS. Among these compounds, curcumin has shown significant photoinactivation effects on cariogenic bacteria in biofilm models [42]. However, curcumin has low stability, so its clinical application requires molecular structure modifications [43]. Nonetheless, BGP extracts are extensively marketed, used worldwide, and present satisfactory biological activities (anti-inflammatory, antimicrobial, and antiparasitic) [44].

This is the first time BGP extracts are tested in antimicrobial photodynamic therapy. Moreover, in the literature, there are no reports on such a high microbial reduction in single-species biofilms (total reduction for *C. albicans* and >5 log_10_ CFU/mL reduction for *S. mutans*) for another PS [11,42,45].

In dual-species biofilms, *C. albicans* viability showed a higher ability to tolerate the photodamage from PDT. This may be due to the increased α-glucans production by *S. mutans*, which surrounds *C. albicans* cells and provides additional protection [46]. This finding also supports the use of *C. albicans*/*S. mutans* dual-species biofilms as a major contributor to oral disease development, such as childhood caries, periodontal diseases, dental pulp infections, and oral mucosal infections [47]. Furthermore, microbial regrowth evaluation shows that PDT potentially helps delay biofilm formation.

The microbial viability reduction in the present study may be supported by ROS production, which induces microorganism injuries and, consequently, death [48,49]. ROS production is also crucial to aPDT efficiency. Singlet oxygen might be the most significant ROS today because it has high oxidative efficiency against prokaryotic cells and low cytotoxicity to human cells [50]. The ROS produced by BGP revealed a higher amount of singlet oxygen (type II reaction) than radical species (type I reaction). The higher concentration of singlet oxygen in the present study might be due to the higher production of triplets, which generate singlet oxygen. These findings may relate to a low extract aggregation in the aqueous solution and a low concentration of dimers formation, which should be further evaluated [51].

Stability in the presence of light (photodegradation) is also a relevant property of PS [52] because rapid photodegradation can compromise biological outcomes [53]. In this context, BGP-AF and BGP-AG showed good stability because photodegradation was not fast after blue LED irradiation using high-intensity energy. Considering the main light sources usually found in dental offices, the present study suggests that the marketed BGP is a highly potential alternative to conventional PS regularly used in aPDT in dental treatments [54].

Propolis is a very complex mixture that contains hydrophobic and hydrophilic substances [55,56]. Its high complexity makes it hard to characterize the product and prepare commercial formulations containing propolis and its derivatives. In this context, it is logical to fractionate the propolis to simplify its composition. Thus, we prepared aqueous propolis extracts. After evaluating the propolis composition [55,56], this study performed an alcoholic extraction of hydrophilic substances followed by the reconstitution of this extract in water [37,38].

For extraction with ethanol, substances with high solubility in ethanol are expected to solubilize in the liquid phase, and substances with low solubility are expected to solubilize in ethanol precipitate [37,38]. For BGP extracts, organic molecules, such as secondary metabolites, highly likely to interact with light at wavelengths in the visible spectrum, are expected to solubilize. Other substances that may compose the soluble fraction of the ethanol extract are peptides and derivatives, polypeptides, carbohydrates, oligosaccharides, fatty acids, and many other organic molecules from these substance classes (including essential oils) [46,48]. The precipitation of macromolecules with low solubility in ethanol, such as proteins and polysaccharides, and a high concentration of waxes and co-related molecules are expected in the insoluble fraction.

The initial separation of BGP extracts was successful, showing the possibility of fractionating and extracting numerous substances capable of interacting with visible light in distinguished substances that absorb light at 450 nm. This initial characterization showed that both extracts have a similar interaction with light and that BGP-AF is more potent because it generated a more intense signal for the extraction and analysis conditions (Table 2). The generation of a more intense signal may explain the better antimicrobial activity. The higher total protein concentration in the BGP-AF extract (Table 3) also contributes to microbiological results because the oxidation of these proteins by light induces cell necrosis and apoptosis [57].

However, BGP fractions showed lower antimicrobial activity in single-species biofilms than BGP crude extracts. This can be explained by the loss of bioactive compounds during fractionation [58] and the biological activity reduction due to the interaction loss between substances in the crude extracts [58,59,60]. Considering that BGP crude extract effectiveness lies in its complex chemical composition [61] because it is vastly marketed and used in its crude form, and the chemical compositions of the two marketed BGP extracts have been extensively addressed in the literature [30,31,62], the present study chose to analyze the chemical composition of BGP. We suggest using crude BGP as a PS in PDT. Moreover, BGP-AF crude extracts have a standardized and highly reproducible composition monitored by characteristic HPLC fingerprint [62]. Phenolic substances, phenolic acids, and flavonoids stand out among the different elements in propolis and are attributed to various biological activities [61,62]. Most correlation studies have shown significance, with higher Pearson’s correlation (r) and determination coefficient (R^2^) values. Therefore, further studies should expect a satisfactory antimicrobial response. The differences between Pearson’s correlation (r) and determination coefficient (R^2^) may be due to variability in singlet oxygen production by each BGP and microbial viability in dual-species biofilm.

A primary concern of using PS in dental treatments is tooth pigmentation and staining of esthetic restorations. Therefore, a BGP pigmentation test was performed in different restorative materials (GIC and composite resin), respecting the pre-irradiation and irradiation times provided in the protocol of this study. The concentration of the used PS does not present any pigmentation risk to dental restorative materials (GIC and composite resin), and it can be safely used repeatedly according to patient needs.

PDT cytotoxicity depends on several interdependent factors, such as physicochemical properties and location of PS (intra- or extracellular), intensity, radiation field, oxygen availability, and the time between PS administration and exposure to light [63]. When assessing cytotoxicity in human oral keratinocytes grown in a monolayer in the present study, the BGP-AF crude extract showed cell viability higher than 70% (Figure 8). This is excellent because in the search for new PS and safer and more effective therapies, the lower the effects on normal tissues and cells, the lower the side effects [64].

The present study shows that aPDT with BGP extract represents a promising alternative to topical antimicrobial therapy, with the advantages of reduced unwanted side effects, low cost, no antimicrobial resistance development, and higher and significant antimicrobial activity compared to literature reports. These advantages encourage further research to confirm the potential application of BGP extracts as a PS in aPDT. Thus, future studies should be performed with more complex biofilm models, such as polymicrobial, active attachment, and intermittent sucrose exposure, before moving to in situ and clinical studies.

## 5. Conclusions

According to the methodology of this in vitro study, BGP-mediated aPDT is a significant and effective alternative to eliminate biofilm cultures of *S. mutans* and *C. albicans*, mainly Apis Flora™ BGP (BGP-AF) extracts. Besides the excellent antimicrobial results, this extract also presented satisfactory outcomes for cytotoxicity and color stability in restorative dental materials. This is the first time propolis extracts were evaluated for antimicrobial photodynamic bioassays. Additionally, these BGP extracts are already commercialized, so applying aPDT in clinical trials and procedures is simple.

## Figures and Tables

**Table 1:** Experimental parameters of the study.

Experimental Group	BGP Concentration	Wavelength (nm)	Irradiance Dose (J/cm^2^)	Intensity (mW/cm^2^)	Pre-Irradiation Time (m)	Irradiation Time in Fractionated Mode (m)
**Biofilm**						
Negative control	N/A	0	0	0	0	0
Light	N/A	450	80	151	0	0
BGP (crude/fraction)	1%	0	0	0	15	18
PDT (crude/ fraction)	1%	450	80	15	15	18

m = minutes. N/A: Not applicable.

**Table 2:** Direct comparison of the signal at 450 nm for the samples measured with a dilution factor of 400×.

BGP-AF (A_450_)		BGP-AG (A_450_)	
Ethanolic	Aqueous	Ethanolic	Aqueous
0.0542	0.2469	0.0432	0.0695

**Table 3:** Concentration of total protein in ethanolic extracts of propolis reconstituted in water. Protein concentration was assessed with the Bradford method.

	Protein (mg/mL)
**BGP-AF**	2.01
**BGP-AG**	1.51

**Table 4:** Summary of two-way ANOVA for single- and dual-species biofilm viability, ROS production, and cytotoxicity.

Source	df	SS	MS	F	*p*-Value
***S. mutans* (single biofilm)**					
Groups	3	176.8	58.92	42,701	<0.0001
Treatment	3	495.6	165.2	119,706	<0.0001
Groups*Treatment	9	437.6	48.62	35,235	<0.0001
***S. mutans* (dual biofilm)**					
Groups	1	18.9	18.9	2328	<0.0001
Treatment	5	599.1	119.8	14,758	<0.0001
Groups*Treatment	5	0.9306	0.1861	22.92	<0.0001
***C. albicans* (single biofilm)**					
Groups	3	49.46	16.49	6556	<0.0001
Treatment	3	373.4	124.5	40,184	<0.0001
Groups*Treatment	9	182.8	20.31	5322	<0.0001
***C. albicans* (dual biofilm)**					
Groups	1	17.58	17.58	77.49	<0.0001
Treatment	5	494.4	98.87	435.9	<0.0001
Groups*Treatment	5	0.759	0.1518	0.6692	0.6476
**ROS**					
Probe	5	1.067 × 10^13^	2.2134 × 10^12^	721.2	<0.0001
Natural	1	3.904 × 10^9^	3.904 × 10^9^	1.319	0.2553
Probe*Natural	5	3.487 × 10^10^	6.975 × 10^9^	2.357	0.0510
**Cytotoxicity**					
Light	1	2508	2508	105.3	<0.0001
Groups	3	104,510	34,837	1462	<0.0001
Light * Groups	3	2782	927.5	39.93	<0.0001

Df = degrees of freedom; SS = sum of squares; MS = mean square; F = MS factor/MS residual; *p* = probability of significance, α = 0.050; * interaction between variable analyses; ROS = reactive oxygen species.

**Table 5:** Summary of correlation studies on singlet oxygen sensor green (SOSG) and log reduction in *S. mutans* and *C. albicans* in dual-species biofilms.

Groups	Variables		*p*-Value	r (Pearson)	R^2^
***S. mutans*—immediate effect**					
BGP-AG	PS + Light (log reduction)	SOSG	**0.0215**	0.8776	0.7701
BGP-AF	PS + Light (log reduction)	SOSG	**0.0047**	0.9432	0.8896
***S. mutans*—regrowth**					
BGP-AG	PS + Light (log reduction)	SOSG	**0.0257**	0.866	0.7499
BGP-AF	PS + Light (log reduction)	SOSG	**0.0023**	0.9602	0.922
***C. albicans*—immediate effect**					
BGP-AG	PS + Light (log reduction)	SOSG	**0.0076**	0.9277	0.8606
BGP-AF	PS + Light (log reduction)	SOSG	**0.0259**	0.8656	0.7493
***C. albicans*—regrowth**					
BGP-AG	PS + Light (log reduction)	SOSG	**0.0198**	0.8828	0.7794
BGP-AF	PS + Light (log reduction)	SOSG	**0.0565**	0.799	0.6384

SOSG: singlet oxygen sensor green probe; r: correlation coefficient; R^2^: determination coefficient. *p*-value < 0.05 represented statistical significance.

## Data Availability

Not applicable.
